# IR Reflectography, Pulse-Compression Thermography, MA-XRF, and Radiography: A Full-Thickness Study of a 16th-Century Panel Painting Copy of Raphael

**DOI:** 10.3390/jimaging8060150

**Published:** 2022-05-24

**Authors:** Tiziana Cavaleri, Claudia Pelosi, Marco Ricci, Stefano Laureti, Francesco Paolo Romano, Claudia Caliri, Bernadette Ventura, Stefania De Blasi, Marco Gargano

**Affiliations:** 1 Centro Conservazione e Restauro dei Beni Culturali “La Venaria Reale”, Via XX Settembre, 18, Venaria Reale, 13473 Turin, Italy; bernadette.ventura@centrorestaurovenaria.it (B.V.); stefania.deblasi@centrorestaurovenaria.it (S.D.B.); 2 Dipartimento di Economia, Ingegneria, Società e Impresa, Università della Tuscia, Via del Paradiso, 47, 01100 Viterbo, Italy; pelosi@unitus.it; 3 Department of Informatics, Modelling, Electronics and Systems Engineering, University of Calabria, Via Pietro Bucci, Arcavacata di Rende, 87100 Cosenza, Italy; marco.ricci@unical.it (M.R.); stefano.laureti@unical.it (S.L.); 4 Istituto di Scienze per il Patrimonio Culturale del Consiglio Nazionale delle Ricerche (ISPC-CNR), Sezione di Catania, Via Biblioteca, 4, 95124 Catania, Italy; francescopaolo.romano@cnr.it (F.P.R.); claudia.caliri@cnr.it (C.C.); 5 Department of Physics “Aldo Pontremoli”, University of Milan, Via Celoria, 16, 20016 Milan, Italy; marco.gargano@unimi.it

**Keywords:** X-ray radiography, IR reflectography, pulse-compression thermography

## Abstract

The potential of any multi-analytical and non-invasive approach to the study of cultural heritage, both for conservation and scientific investigation purposes, is gaining increasing interest, and it was tested in this paper, focusing on the panel painting *Madonna della Tenda* (Musei Reali, Turin), identified as a 16th-century copy of the painting by Raffaello Sanzio. As a part of a broader diagnostic campaign carried out at the Centro Conservazione e Restauro, La Venaria Reale in Turin, Italy, the potential of the combination of X-ray radiography, pulse-compression thermography, macro X-ray fluorescence, and IR reflectography was tested to investigate the wooden support and all the preparatory phases for the realization of the painting. The results of the optical microscopy and SEM/EDS analyses on a multi-layered micro-sample were used for a precise comparison, integration, and/or confirmation of what was suggested by the non-invasive techniques. Particularly, the radiographic and thermographic techniques allowed for an in-depth study of a hole, interestingly present on the panel’s back surface, detecting the trajectory of the wood grain and confirming the presence of an old wood knot, as well as of a tau-shaped element—potentially a cracked and unfilled area of the wooden support—near the hollow. The combination of radiography, macro X-ray fluorescence, Near Infrared (NIR), and Short Wave Infrared (SWIR) reflectography allowed for an inspection of the ground layer, imprimitura, engravings, and underdrawing, not only revealing interesting technical-executive aspects of the artwork realization, but also highlighting the advantages of an integrated reading of data obtained from the different analytical techniques.

## 1. Introduction

Previously attributed to Raffaello Sanzio, the panel painting *Madonna della Tenda*, belonging to collections of the Musei Reali in Turin, has been instead historically identified as a copy imitating Raffaello Sanzio—the original work is displayed at the Alte Pinakothek in Munich, Germany—dated to around 1525–1550, and made by a painter that worked in a central Italy workshop. The scientific investigations on this painting were part of wider interdisciplinary research that involved numerous professionals and institutions, whose outcomes were presented during the temporary exhibition “Sulle tracce di Raffaello nelle collezioni sabaude” (Turin, Musei Reali–Galleria Sabauda, first opening 30 October 2020 and closing 2 June 2021) and reported in the exhibition catalogue [1].

Some of the scientific analyses carried out were preliminary to the conservation treatment on the artwork, realized by the Centro Conservazione e Restauro, La Venaria Reale (CCR) for an exhibition focused on the critical fortune of the Raffaello’s school and the old master paintings in the Savoy collection. The scientific campaign was firstly addressed to verify the state of preservation of both the wooden support and the pictorial layers, the latter having visible issues near the Child’s right eye, precisely in correspondence of a hole on the back surface of the panel. Moreover, some seals and the inscription “*Quadro del divino Rafaele Sanzio/D’Urbino*” are visible on the back panel’s surface. The artwork’s conservation history and the chance to have a technical comparison with the authentic counterpart in Munich, have offered numerous stimuli to deepen different technical and material aspects that were investigated through an interdisciplinary collaboration and a multi-analytic approach.

Thanks to the fruitful collaboration between the physics department of the University of Milan and the department of informatics, modeling, electronics, and systems engineering (DIMES) of the University of Calabria, both in Italy, the conservation issues linked to the panel and hole were investigated through X-ray radiography (XR) and pulse-compression thermography (PuCT), both of which are imaging techniques whose effectiveness in non-destructively investigating the morphological and physical features of a panel painting’s inner structure is well known [2,3,4].

XR is, in fact, a well-established technique that allows conservators and art historians to acquire a global view of the structure of an object, from the back of the support to the outer painting layers, such as the assembling technique, the type of and state of preservation of the wood, primer, and pictorial layers, and potentially, of the underdrawing [5,6,7,8,9]. However, it requires a particular setup, due to the risk of exposure to X-rays.

All the radiographic data are important, and conservators and art historians are usually very interested in the issues related to the support, considering the information coming from the paint layers precious indications to distinguish different stages of a painting, and to help infer the work of different artists’ hands and past restorations. It must be stressed that the combined information of the three-dimensional structure of a painting can lead to difficulties in interpretation when isolating information from a particular layer is needed. This segmentation can be obtained using different penetrating radiation, such as near-infrared (NIR), shortwave infrared (SWIR), and mid-wave infrared (MWIR) [10,11,12,13]. The first two are performed using the reflected infrared spectra from the painting surface, usually up to the preparation layer, while the latter is carried out by measuring the emitted infrared by the outer and the inner layers of the painting after a thermal stimulus. By combining NIR and SWIR multiband reflectography, it is possible to differentiate the underdrawing and pigments, highlighting specific features related to the drawing technique in order to map restorations, thanks to the different IR absorption properties of the pigments.

It is worth noting that the limitations of the penetration due to the preparatory layers can be overcome using IR thermography. IR thermography, either active or passive, is a well-established method for monitoring, assessing, and evaluating the integrity of different materials [14,15], including historical multi-layered structures such as frescos, canvas [16], and panel paintings [17], to mention a few [18,19,20]. Usually, the use of external heating sources, i.e., active thermography, provides more insight into the inspected item’s stratigraphy with respect to the use of passive thermography [21]. In fact, the heating source, e.g., a halogen lamp, is switched on/off following a known reference waveform in time, e.g., step-heating, sinusoidal modulation, or single pulse, to which analytical/empirical post-processing, and different data visualization methods can be applied for extracting useful information [22]. However, when dealing with irreplaceable items, such as the here-inspected panel painting, heating must be used carefully so as to avoid any potential for thermochromism and/or cracking due to exceeding thermal stress.

To circumvent the mentioned problems while maintaining good inspection capabilities, some of the present authors have demonstrated that the use of low-power light emission diodes (LEDs) in the visible range, whose on/off state is modulated via a pseudo-noise waveform, is a reliable method for the safe inspection of masterpieces. By using such a modulation, it has been shown that it is possible to retrieve valuable information, e.g., thermal signatures from groutings, detachments, and other inner inhomogeneities, with a gentle rise in the sample surface’s temperature <2 °C in 82 s [23,24]. This is made possible by the contextual use of the so-called pulse-compression (PuC) as a post-processing technique, which allows for replicating a fictitious pulsed thermography measurement from the pseudo-noise recorded data. For the mentioned reasons, PuC thermography (PuCT) has been used to safely evaluate the pictorial and sub-pictorial layers of the inspected item. The interested reader is referred to Laureti et al. [25], Wu et al. [26], and Silipigni et al. [27] for further information on the PuCT theoretical background.

In this work, the integrated results of the above-mentioned methods were additionally compared to the elementary maps results of the macro-X-rays fluorescence (MAXRF) examinations through a collaboration with the Institute of Heritage Science of the National Research Council (CNR-ISPC) section of Catania, Italy. Finally, the study of the underdrawings and other possible preparatory phases (such as engravings) was carried out through high-resolution infrared reflectography (IRR) in the NIR and SWIR band [28,29,30], combined again with the information of the radiographic image and the MA-XRF maps, allowing a complete representation of all the preparatory phases of the painting to be inferred. The reading of a stratigraphic sample is reported here for comparison and as a tool for confirming the results from non-invasive analyses.

## 2. Materials and Methods

### 2.1. The Panel Painting Madonna Della Tenda: Technical Features

**Figure 1:**
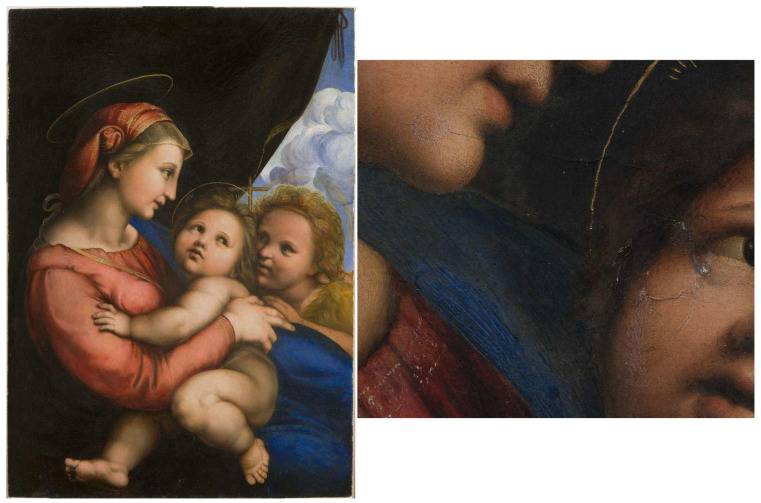
The panel painting *Madonna della Tenda* (Turin, Musei Reali–Galleria Sabauda, Inv. 271) and a detail of the state of preservation.

The panel painting *Madonna della Tenda* depicts the Virgin and Child with Saint John (Figure 1). As described in the technical contribution in the exhibition catalogue [31], it is made on a poplar wood support, having dimensions of 79 × 56 cm, with an average thickness of about 2.5 cm, varying from 2 cm on the bottom left margin to 2.8 cm in the central part. The support is also affected by a significant curvature on its central part, having a maximum deflection of about 1.5 cm, as well as by slight deformations. It has no crosspieces for deformation control, and a slight warping is noticeable. The preparatory and pictorial layers are found on the face of the table facing outwards.

**Figure 2:**
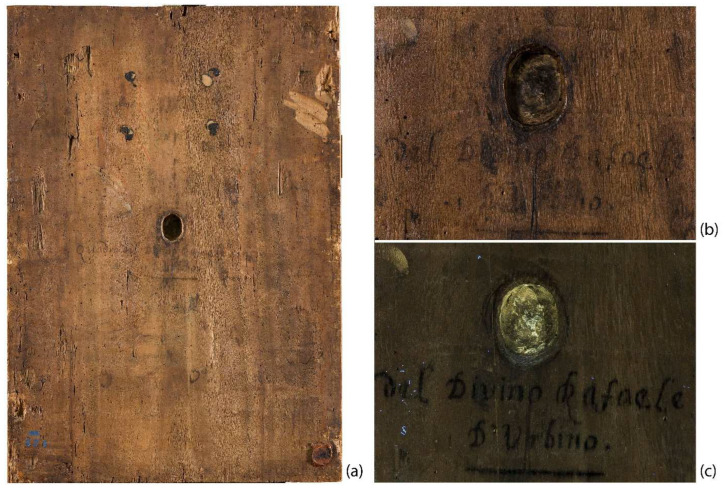
Back panel surface where the hollow and the handwritten inscription are visible (**a**); detail of the hollow under visible (**b**) and UV light (**c**).

On the painting’s back surface, there are visible signs of woodworking and recessed parts, and a hollow caused, at first analysis, to the loss of a knot, subsequently widened: the edges of the hole are dug and engraved, showing traces of adhesive substances on the internal walls (Figure 2). In correspondence to the hole, the wooden support is so thin that it provoked deformations and fractures on the front of the painting (Figure 1). Additionally, on the back of the panel, signs of several changes of ownership and re-location for restoration purposes can be found—from the Porporati seal of Piossasco, to traces of inventory numbers, and the Milan customs wax seal. In the middle of the back, a black ink handwritten inscription is clearly visible: “*Quadro del divino Rafaele Sanzio D’ Urbino*” (Figure 2).

### 2.2. Analytical Methods

The investigation methods applied at the CCR for studying the painting followed a well-established analytical protocol based on subsequent analytical phases that included traditional and state-of-the-art techniques. With the aim of providing the best response to the questions raised by conservators and art historians, the techniques available at the CCR and also provided by the valuable above-mentioned scientific research collaborations were applied.

As part of a wider diagnostic campaign, the analytical methods discussed here are those which have proved most useful for studying the characteristics of the wooden support and the preservation problems linked to it, as well as for the study of the early stages of the artwork realization, from preparation layers to engravings and underdrawing. As can be seen from the descriptions below, all are imaging methods, the results of which, linked together, have allowed for a thorough non-invasive reading of the painting’s stratigraphy, the result of which was only confirmed by a later (micro-)invasive analyses on stratigraphic micro-samples.

#### 2.2.1. Technical Photography

Visible diffuse light photography (Vis) was achieved by using two 800W Ianiro Varibeam Halogen lamps (Ianiro, New Taipei City, Taywan), placed at an angle of approximately 45° to the surface, and with the aid of umbrellas for light diffusion purposes. Photographs were taken using a Nikon D810 Full-Frame DSLR camera (CMOS 7360 × 4912 pixels).(Nital Spa, Moncalieri, Turin, Italy)).

The UV-induced visible fluorescence (UVF) was acquired via a Nikon D810 full-frame DSLR camera (CMOS 7360 × 4912 pixel) and a Hoya UV-IR Cut filter (Hoya, Tokyo, Japan), irradiating the painting with two UV Labino^®^ lamps (365 nm-emission peak). The NIR reflectography was performed using a Nikon D810 full-frame DSLR camera (CMOS 7360 × 4912 pixel), modified to extend the sensor sensitivity range up to 1000 nm, and with a B+W 093 (87c) filter, illuminating the painting using Ianiro Varibeam Halogen 800 W lamps (Ianiro, New Taipei City, Taywan) with indirect diffused light.

Image processing was carried out by means of Adobe Lightroom and Adobe Photoshop software (Version C6S) and included a color correction conducted by inserting a 24-color ColorChecker Classic reference in the field of view.

#### 2.2.2. X-ray Radiography (XR)

XR was performed using the traditional RX film technique, a General Electric Eresco 42MF4 X-ray source (200kV max, 4.5 mA) (General Electric, Boston, MA, USA), and AGFA D4DW plates (General Electric, MA, USA) of 30 × 40 cm in size. In addition to the exposure tests, six plates were exposed to cover the entire painting’s surface with an adequate overlap, setting the tube tension to 43 kV for 1 min. The painting was positioned vertically in front of the X-ray source at a 150 cm distance. The plates were then developed in a darkroom at a temperature of 20 °C for 6 min, and subsequently digitized and merged using Adobe Photoshop.

#### 2.2.3. Pulse-Compression Thermography (PuCT)

**Figure 3:**
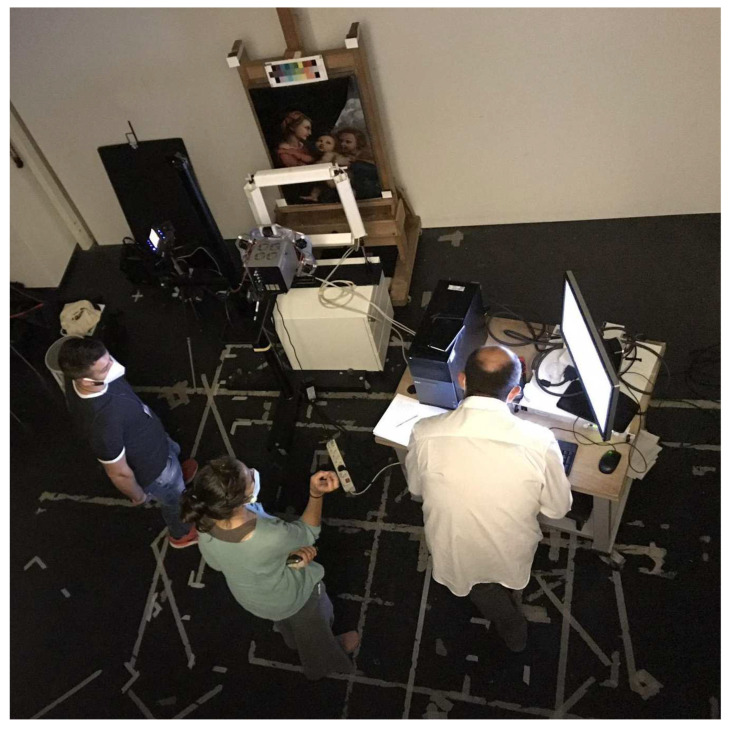
A photograph during PuCT acquisition. The LED system and the thermal camera are placed in front of the inspected painting.

The here-employed PuCT setup was the same used in other works, see for example [3,32,33]. In particular, a National Instrument 1433 Camera Link Frame Grabber and National Instrument PCI-6711 AWG board (National Instruments company, Austin, TX, USA) was used to acquire thermograms from a Xenics Onca-MWIR-InSb IR (XenICs, Leuven, Belgium-320 × 256 pixels, 3.6–4.9 μm) at 40 FPS and to modulate the on/off state of a TDK Lambda GEN 750 W power supply (TDK Corporation, Tokyo, Japan), respectively. In this way, 8 LED chips (Tesfish, Shenzen, China—6500 K, 4500 LM) were driven at 200 W so as to emit light following a Legendre code pseudo-noise excitation of order equal to 31 for an overall heating stimulus duration of 62 s—the reader is referred to [34,35] for an in-depth mathematical explanation on the correct use of such code in PuC applications. The LED system and the thermal camera were placed on the same side at about 50 cm from the inspected surface, so that the resulting inspection was performed in reflection mode. A desktop computer was used to store the data using an in-house routine developed in LabVIEW. A picture of the PuCT setup is shown in Figure 3. Note that both the camera and the LED were moved after each measurement—the limited field of view and relatively low pixel resolution of the thermal camera are used because the whole painting could not be inspected otherwise. A mosaic was then reconstructed by sticking each measurement next to the others. Finally, note that all the reported results are shown using a grey colormap, where the darker pixels are related to higher temperature/emissivity.

#### 2.2.4. Macro X-ray Fluorescence (MA-XRF)

MA-XRF was performed via a mobile X-ray scanner, used in situ at the CCR and developed by the XRAYlab of the Institute of Cultural Heritage Sciences, National Research Council (ISPC-CNR), and by the South National Laboratories, Institute of Nuclear Physics (LNS-INFN), Catania, Italy.

The scanning system allows for analyzing large pictorial surfaces up to 110 × 70 cm^2^. The time taken for inspecting the whole painted area was 2.5 h, with a lateral resolution of 1000 µm at the maximum scanning speed of 100 mm/s [36].

This innovative real-time technology allows for a continuous scanning of the surface to be performed, instead of using the conventional step-by-step mode. All the images of the chemical elements composing the pictorial layers are elaborated in live mode, and its distribution on the support is visualized in real-time during the scanning.

The primary X-ray beam is generated by an Rh-anode tube (50 kV maximum voltage and 0.6 mA maximum current) and it is focused on the sample by a polycapillary optics coupled with the X-ray source.

A time list mode (TLIST), implemented in a multi-detector system composed of two 50-mm^2^ silicon drift detectors (SDD) (Ketek GmbH, Munich, Germany) with a 160 eV spectral energy resolution at 5.9 keV, is used to detect the emitted fluorescence radiation. The detection system allows for a time/pixel in the ms range and high counting statistics.

A fast-fitting procedure (max processing speed: 7000 spectra/s) is applied to all pixel spectra generating on-the-fly elemental images. During the scanning, the movement of the *z*-axis adjusts the measuring head/surface distance, monitored in real-time by a laser sensor, to follow, if present, the non-flat surface of the sample [37,38,39,40,41].

The whole area of the painting (consisting of c.a. 537 × 802 pixels) was analyzed with a scanning speed of 70 mm/s, a dwell time/pixel of 15 ms, and a lateral resolution of 1 mm. The measurement time was 1.8 h, and the primary X-ray beam voltage was set at 50 kV, with a current of 600 µA.

#### 2.2.5. High-Resolution Shortwave Infrared Reflectography (HR-SWIR)

The high-resolution shortwave infrared reflectography (HR-SWIR) was performed with a spherical scanning system [42] based on an InGaAs camera (Xenics Xeva 1.7–640, 640 × 512 elements, spectral sensitivity: 1000–1700 nm) (Xenics, Leuven, Belgium) mounted on a spherical motorized head, with a linear stage for refocusing. A total of 2200 images were acquired and merged to obtain a final image with a spatial resolution 20 pixel/mm. Two halogen lamps, with indirect and diffusing screens, were used for the lighting.

#### 2.2.6. Optical Microscopy (OM) and Scanning Electron Microscopy with Energy-Dispersive X-ray Spectroscopy (SEM/EDS)

The multi-layered sample taken from the Virgin’s blue mantle (overall, eight multi-layered samples were taken from the painting for conservation and scientific investigation purposes; the sample reported here is representative and functional for a comparison with the results of the imaging techniques that are the focus of this manuscript) was observed and photographed under visible light using an Olympus SZX10 stereomicroscope equipped with an Olympus Color View I digital camera (Olympus Italia S.r.l., Milan, Italy). After being arranged as a cross section, the sample was photographed under visible and ultraviolet light using an Olympus BX51 minero-petrographic microscope equipped with an Olympus DP71 digital camera (Olympus Italia S.r.l., Milan, Italy). In both cases, image acquisition and processing were performed using the analySIS FIVE proprietary software (Version 2005). The cross section was then analyzed in back-scattered electron mode (BSE) using a Zeiss EVO60 scanning electron microscope equipped with a lanthanum hexaboride (LaB6) cathode (Carl Zeiss Microscopy GmbH, Jena, Germany) and a silicon drift detector (SDD), coupled with a 40 mm^2^ Oxford Ultim Max EDS microprobe (Oxford Instruments NanoAnalysis, Gometz la Ville, France) for semi-quantitative elemental analysis. It was coated with a nanometric layer of carbon and analyzed in high vacuum mode, using an accelerating voltage of 20 kV and a pressure of 10^−5^ Pa.

## 3. Results and Discussion

### 3.1. Wooden Support: The Hole on the Painting’s Back Surface and State of Preservation

**Figure 4:**
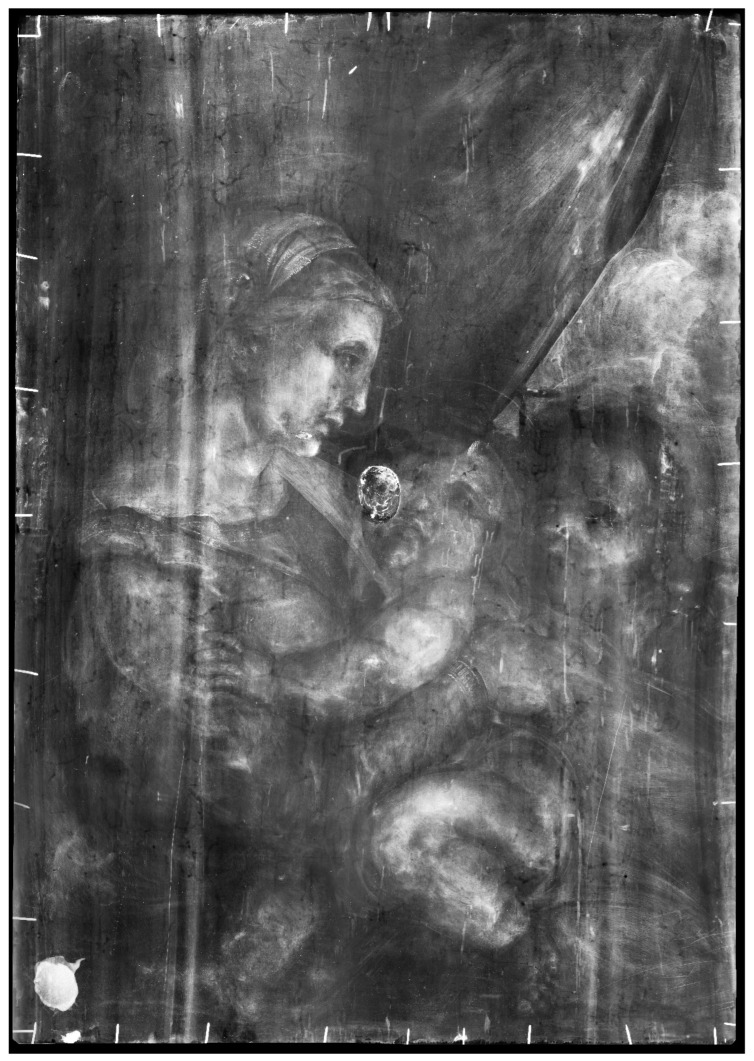
XR of the painting showing the pattern of wood grains of the support (much less noticeable in the central stripe) and the numerous woodworm tunnels following the pattern of the wood fibers.

XR showed that the panel was made out of a single poplar wood board, recognizable by the typical radiographic pattern of the wood grain (Figure 4). The presence and the nature of the hole on the painting’s back surface—and consequently, the features of the wood grain, the woodworm tunnels, and other peculiar characteristics of the wooden support—have been fully investigated. For ease of reading, the results discussed below are summarized in Table 1. The table also shows the synergy and complementarity of the analytical techniques used when studying the artwork (Table 1).

**Figure 5:**
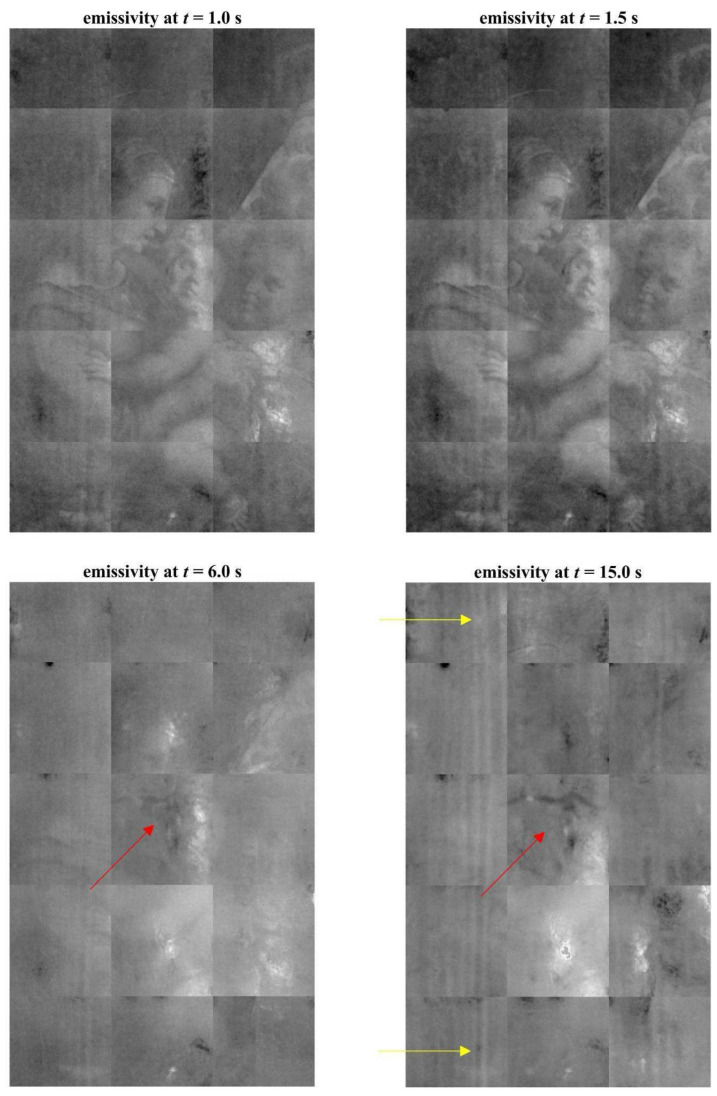
A series of thermograms at different time instants obtained by PuCT. A “Tau-shaped thermal signature is visible, starting from *t* = 6 s (red arrow). As a reference, the yellow arrows point out a single vertical wood grain signature. Note that other quasi-parallel grains are also well defined all across the inspected surface.

**Figure 6:**
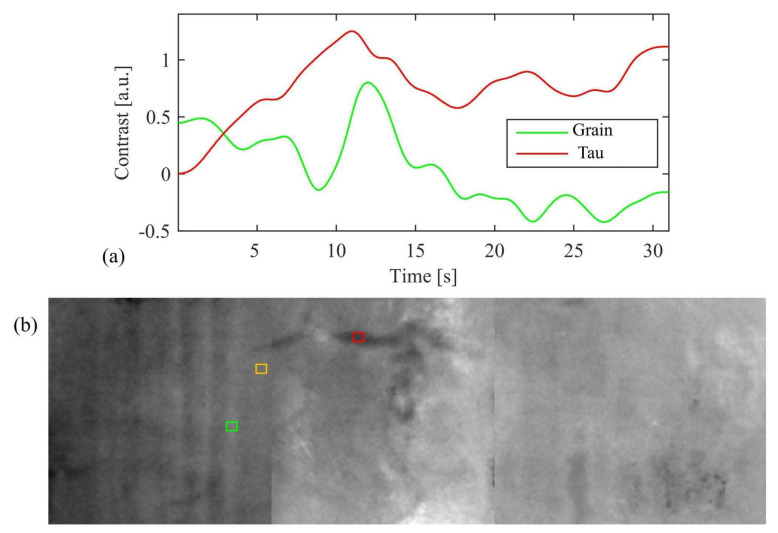
(**a**) Thermal contrast for the grain (green) and tau (red) pixel areas as a function of time. A maximum is reached for both quantities at *t =* 13 s; (**b**) a thermogram showing the investigated area at *t =* 13 s, marked to highlight the areas employed for computing the thermal contrast, i.e., tau within the red square, wood grain within the green square, and sound within the orange square.

The hole is so deep that it reaches a few millimeters from the painted surface. It has engraved edges (see Figure 2) and is treated on the bottom with colophony and beeswax, probably with an insulating function. The material identification is based on the Py-GC/MS analysis conducted by the chemistry department of the University of Turin (Prof. Dominique Scalarone and Dr. Chiara Riedo). The combined results of XR and PuCT made it possible to detect the original presence of a small knot, largely removed. In XR the wood grain is particularly evident in the side portions, but much less noticeable in the central stripe (Figure 4). In PuCT, the widening of the wood grain inside the thickness of the board can be observed around the hole. Moreover, a tau-shaped element is clearly visible near the hole using both techniques. Figure 5 shows a series of thermograms at increasing time instants obtained using PuCT. The thermal signature from deeper layers becomes visible as time elapses—the pictorial layer tends to disappear at a time value equal to 6 s, while the details belonging to the inner wooden panel simultaneously appear more and more clear. Interestingly, the tau-shape, hereafter referred as tau, thermal signature is visible from *t =* 6 s (see red arrows) to *t =* 15 s. In addition, a series of vertical strips become evident during the same time interval (yellow arrow); these correspond to the wood grains, which are also visible in the XR analysis (see Figure 4). The depth of the tau from the inspected surface, i.e., the pictorial layer, can be inferred by comparing the thermal contrast obtained from a pixel area of the tau, and that obtained from an area of the wood grain. These 4 × 4 pixels areas are highlighted using red- and green square markers respectively, while orange has been used to mark the sound area employed as a reference for computing the thermal contrast (see Figure 6b), with the thermal contrast defined as:

(1) $$\begin{array}{c} Contrast_{Tau}\left(t\right)=\frac{Tau\;\left(t\right)-sound\;\left(t\right)}{sound\;\left(t\right)},\\ Contrast_{wood}\left(t\right)=\frac{wood\;\left(t\right)-sound\;\left(t\right)}{sound\;\left(t\right)}, \end{array}$$

with *wood* (*t*), *Tau* (*t*), and *sound* (*t*) being the averaged temperature at the selected grain, tau, and sound areas, respectively. The line plots in Figure 6a show the obtained contrasts within the evaluated time interval; the two maxima are reached at about 12 s, indicating that the tau is located at the same depth of the wood grain. In other words, the tau is a feature belonging to the support panel. Interestingly, the tau signature appears darker than the surrounding area (see Figure 6b) where the thermogram at 12 s is imaged, meaning that its temperature is higher than that of the other pixels. Commonly, this happens when a material with a lower thermal capacity is found, a good example being delaminations with air trapped within the preparatory layer and the wood support. This suggests that the tau is potentially a cracked and unfilled area of the wood support.

Interestingly, the tau is located geometrically close to the hollow area visible from the back surface of the panel. It is worth investigating whether more information on the nature of the tau can be inferred using the PuCT analysis of the back surface of the panel.

**Figure 7:**
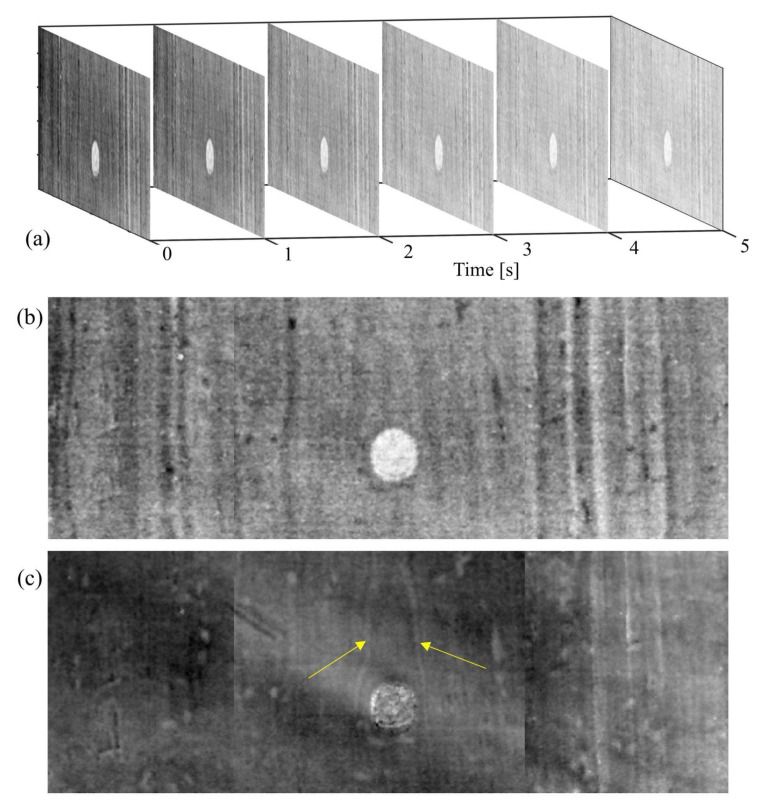
(**a**) a series of thermograms at different time instants acquired from the back panel surface using PuCT; (**b**) same as (**a**), but for a single *t =* 4 s; (**c**) image obtained using the time-phase feature, with yellow arrows pointing towards the wood grain surrounding the hollow area.

To this aim, Figure 7a shows a series of thermograms at different time instants, whereby both the hollow wood area and the wood grains appear clearly. To gain more insight on the back surface of the support, Figure 7b,c depicts the emissivity at *t* = 4 s and the time-phase feature at *t* = 1 s, respectively. Both the imaged features show an advanced state of degradation of the inspected surface, where woodworm holes are also visible, especially in Figure 7c. Interestingly, the number of visible grains is considerably higher at both the left- and right-end sides, compared to that visible nearby the hollow part. Moreover, the most visible wood grains near the hollow follow a smooth trajectory around it, suggesting/confirming that the hollow part could have been a wood knot. This suggests in turns that the tau might have been generated due to the fragile nature of this part of the panel support, which is further confirmed by the potential loss of the knot.

As a visible result, in correspondence with the hole, the wooden support is so thin that it has caused deformations and fractures on the pictorial film; moreover, in correspondence with the surface along the tau, other points of fragility were found, such as the one on the Virgin’s chin (see Figure 1).

**Figure 8:**
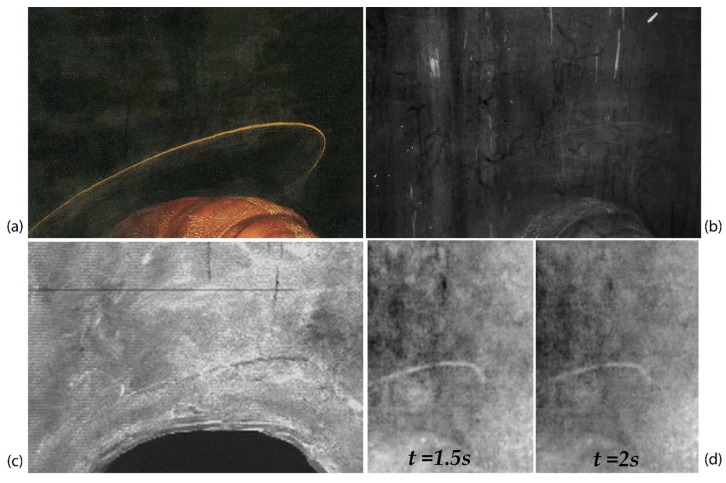
Vis, detail of the green curtain painted with a copper-based green pigment (**a**); XR showing the woodworm tunnels and the more radio-opaque vertical lines (**b**); MA-XRF distribution map of copper showing the lower Cu-signal along the vertical lines (**c**); PuCT thermograms at 1.5 s and 2 s when the dark vertical lines became whiter (**d**).

As observed in XR (see Figure 4), in many cases, the numerous woodworm tunnels follow the pattern of the wood fibers and are visible as a darker and less radio-opaque trace due to the lack of wooden material, whereas other vertical signs are more radio-opaque. Figure 8 shows an example of these signs in correspondence to the green curtain, which is painted with a copper-based green pigment. Along the more radio-opaque lines, a lower signal of copper is detected, as evidenced by the greyscale MA-XRF distribution map of copper (Figure 8c), where the lines appear less bright, while no other elemental differences are detected using the same technique. In the same area, a series of thermograms at different time instants acquired from the front panel surface using PuCT show these vertical lines as dark lines, becoming whiter as time elapses, suggesting that these have been filled and/or restored (Figure 8d). Therefore, these lines could be cracks filled with some radio-opaque compound, not better identified, that probably involves only the upper layers: in correspondence with such signs, in fact, the painting exhibits some micro-cracking, suggesting that they are probably on the surface of the painting and not inside the wooden panel, as is the case for the tunnels caused by worms.

**Table 1:** Summary of the results from XR, PuCT, and MA-XRF analyses of the wooden support.

Wooden Support	XR	PuCT	MA-XRF
Wood grains (general view)	The wood grain, typical of poplar wood, is particularly visible in both the left- and right-end sides of the panel, and are much less noticeable in the central slice containing the hole.	The number of visible grains is considerably higher at both the left- and right-end sides, compared to those visible near the hole.	Not detectable
Wood grains around the hole	Not evident	The wood grain inside the thickness of the board widen around the hole: the most visible wood grains near the hollow follow a smooth trajectory around it, suggesting/confirming that the hollow part could have been a wood knot.	Not detectable
Tau-shaped element near the hole	Clearly visible	Clearly visible; it is located at the same depth of the wood grain, thus belonging to the support; it appears darker than the surrounding area (its temperature is higher than that of the other pixels/it has lower thermal capacity, so it is potentially a cracked and unfilled area of the wood support).	Not detectable
Woodworm tunnels	Visible as radio-transparent traces in the central part of the panel: in many cases they follow the pattern of the wood fibers.	Visible	Not detectable
Other vertical signs (area of the green curtain)	Visible as more radio-opaque vertical traces, since filled and/or restored.	Visible as dark lines, becoming whiter as time elapses, in the thermograms acquired at different times from the front panel surface, Figure 8-suggesting that these have been filled and/or restored; filler probably involves only the upper layers.	Only visible in the Cu-distribution map (a lower signal in the distribution of copper make the vertical signs visible).

### 3.2. Preparation Phases: From the Ground to the Underdrawing

The OM and SEM/EDS analyses on the multi-layered sample taken for the study of the executive technique confirmed what can be hypothesized from the non-invasive imaging techniques. Again, for ease of reading, the results discussed below are summarized in Table 2, showing the complementarity of the analytical methods.

**Figure 9:**
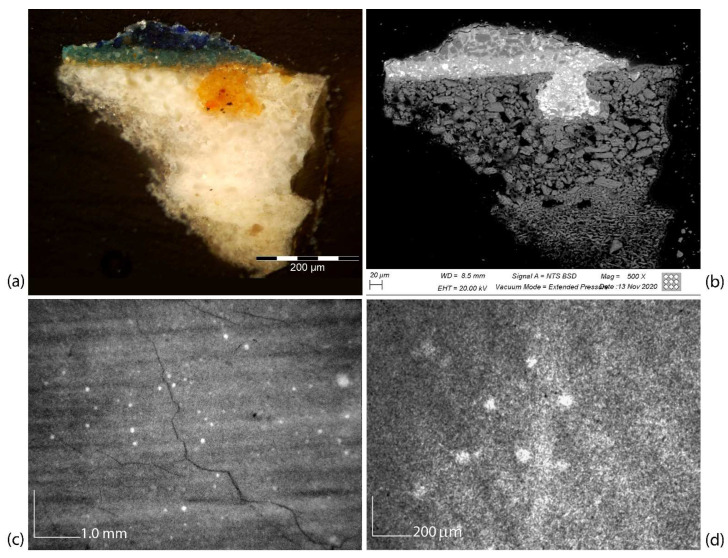
Microscopic images of a cross section of the Virgin’s blue mantle sample in OM (**a**) and SEM/BSE (**b**), showing a yellow-orange rounded inclusion of pigmented imprimitura in the plaster preparation. Above this, two pictorial layers—the first in azurite, the second in lapis lazuli—render the color of the blue mantle. The size of the imprimitura inclusions in the plaster is fully compatible with the enlarged radiographic details at 55× (**c**) and 220× (**d**).

First of all, the cross section shows that the wooden panel is prepared on the front with layers of plaster and animal glue of white color; the plaster layer is followed by a thin layer of a yellow-orange imprimitura, made of lead white and ochres (Figure 9a): this layer is applied with a brush and is particularly transparent so as to allow the drawing to be seen. Note that this is in line with what is reported in the contribution dedicated to the technical study of the work in the exhibition catalogue [31].

Looking at the painting’s XR at high magnification, numerous inclusions of more radiopaque material can be seen, appearing as the widespread presence of small, irregular white spots (Figure 9c,d). Comparing the cross section images (Figure 9a,b) with the radiographic details, it is possible to attribute their presence to bubbles formed during the preparation and mixing of the plaster; these bubbles during the smoothing of the preparation are opened and can be filled by the imprimitura, thus showing very radiopaque and diffuse dots on the whole surface of the painting. The average size of these inclusions corresponds to approximately 100 µm.

**Figure 10:**
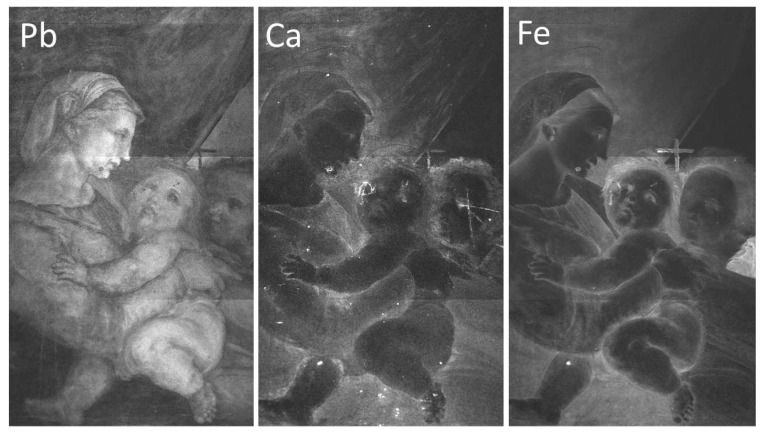
MA-XRF distribution maps of lead, calcium, and iron.

MA-XRF technique confirms the use of lead-, calcium-, and iron-based pigments for the preparation phases of the panel (ground and imprimitura), as evidenced by their distribution extended on the whole support shown in Figure 10. However, the information provided by MA-XRF is integrated along with the pictorial stratigraphy up to the preparatory layer and the contribution of Pb, Fe, and Ca signals, shown in Figure 10, comes not only from the preparatory layer but also from the pigments or compounds that compose the upper layers. In particular, the lead white pigment was also used for obtaining the characters’ skin tones and the highlighting effect on the robes and curtain. In the same way, iron-based pigments, presumably ochres, were used for painting the brown tones such as the characters’ hair. Finally, calcium-based pigments are not present in the superficial pictorial layers, except for in small regions affected by restorations, such as grouting or fillings. In addition, due to the chemical nature of the pigments, the stratigraphic structure of the painting can make the detection of low-energy fluorescence lines difficult, especially when the low Z-elements, such as calcium, are present in the deeper layers. Indeed, the emitted fluorescence is almost completely absorbed in the thicknesses of the upper layers composed of heavier elements, such as lead or copper. This absorption effect is visible in the maps of Figure 10, observing the Ca and Fe signals coming from the preparation; these are strongly absorbed in the upper layers characterized by Lead white-based areas of paint.

**Figure 11:**
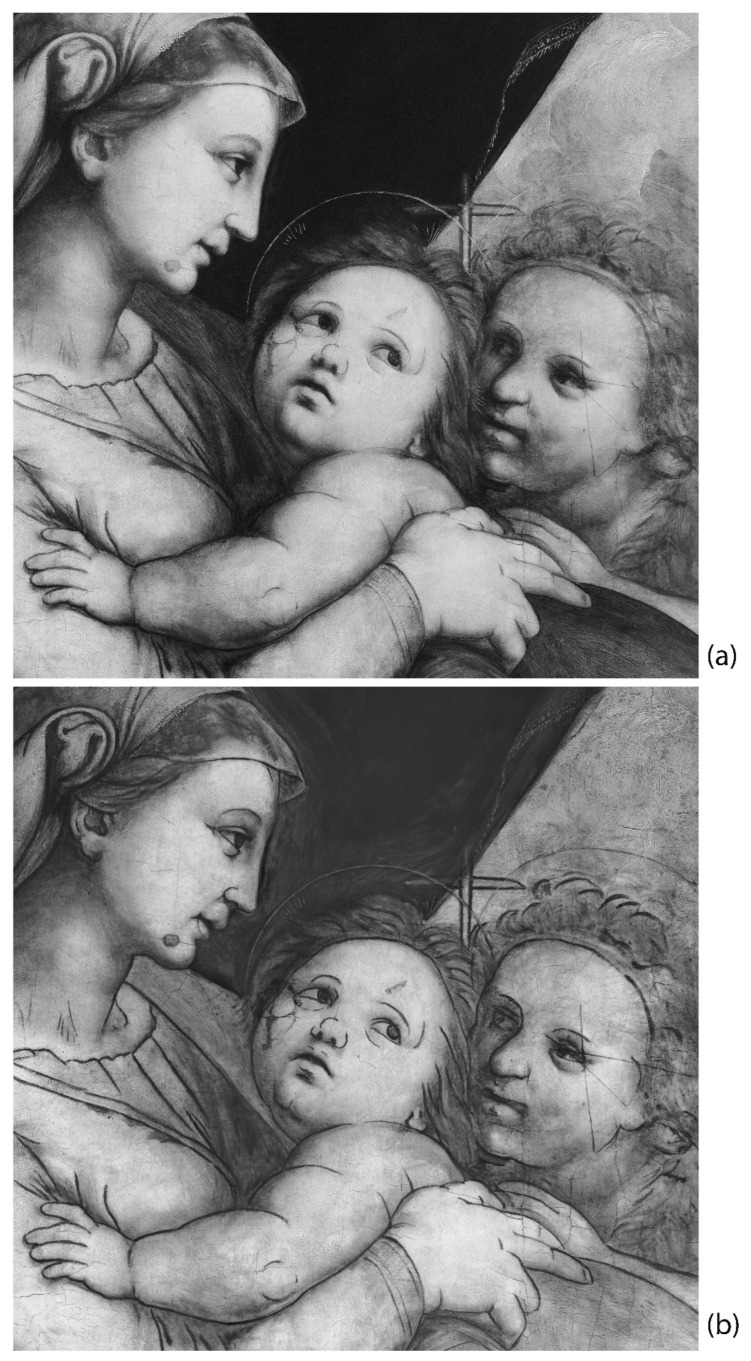
NIR (**a**) and HR-SWIR (**b**) reflectography image comparison showing the difference in revealing the underdrawing made with a brush and a carbon black ink.

**Figure 12:**
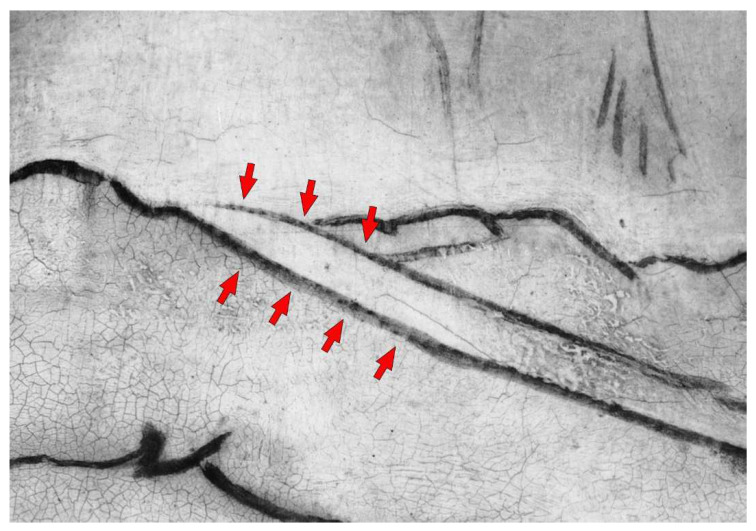
HR-SWIR detail showing the traces of punched dots, indicated by the red arrow, in correspondence to the neck of the Madonna.

The underdrawing is clearly visible using infrared reflectography, as shown in Figure 11. The NIR image (Figure 11a) allows for a view of the main traces, but it is still limited by the low ability for penetrating all the pigment layers. HR-SWIR reflectography (Figure 11b) is instead able to highlight all the details, even in those areas with darker or copper-based pigments: it shows a very detailed, clean, and skillful stroke by means of an underdrawing made with a brush and a fluid carbon black ink. Additionally, it allows for hypothesizing the presence of a preparatory cartoon transferred using punched dots and carbon powder. Some punched dots are barely visible in correspondence to the underdrawing (Figure 12), probably because of the use of a brush for their removal and because they are diluted in the main underdrawing traces completed freehand. Lastly, the artist refined the drawing by adding shading and other indications, which helped him in the painting phase as a guide for the execution of the drapery of the clothes and details of the figures.

**Figure 13:**
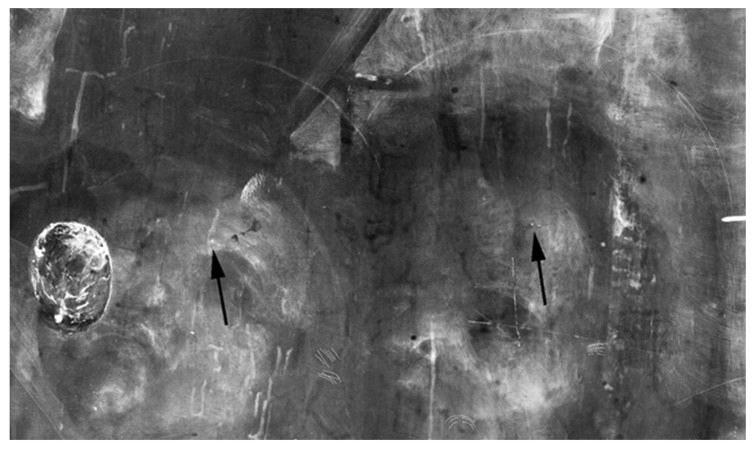
XR detail, where the signs of metal point engravings and needle points are evident in the tracing of the two haloes by means of a drawing compass.

Besides the traces of underdrawing made with black ink, signs of metal point engravings and needle points are also present as a consequence of the construction of the figures’ haloes, made using a compass (Figure 13), presumably drawn on the plaster layer. As reported in the technical study of the artwork, the haloes were subsequently gilded and then repainted again with gold during restorative interventions [31]. The traces of these haloes are also visible as a more intense signal in the MA-XRF distribution maps of lead, probably due to the greater thickness of imprimitura inside the groove left by the compass. On the other hand, in the distribution maps of calcium and iron, the form of the haloes appears as a less intense signal, due to their absorption in the upper layer characterized by the presence of gold.

## 4. Conclusions

The high potential of non-invasive imaging techniques applied to study a panel masterpiece has been thoroughly investigated. Specifically, the combination of PuCT, XR, MA-XRF, and IR reflectography was tested to investigate the wooden support and all the preparatory phases for the realization of the painting—from the ground to the underdrawing—aiming at studying in-depth the specific aspects of the artistic technique and for understanding both the nature of the hole on the panel back surface and the overall state of the preservation of the artwork.

XR and PuCT were particularly useful to study the wood grain characteristics and to detect defects, such as tunnels, groutings, and cracks in the panel support. PuCT allowed for an inner view of the board’s thickness, resulting in the clear observation of the wood grain widening around the hole, confirming that the hollow area could have been a wood knot. As it was also subsequently widened and engraved, the wooden support in correspondence of the hole is so thin that it provoked deformations and fractures on the front of the painting, which in fact have been restored, with fillings that are clearly visible in XR and MA-XRF.

Furthermore, XR and PuCT allowed for the detection of a tau-shaped element near the hole: PuCT aided in its localization in the thickness of the board, confirming it as an element belonging to the wood panel. The tau’s analytical answer in both techniques suggested that this part was a cracked and unfilled area, presumably generated due to the fragile nature of this part of the board. Again, concerning points of fragility and the state of the preservation of the panel, a clear view of the woodworm tunnels was possible using both the techniques: in the high-resolution XR image, the tunnels visible as radio-transparent traces follow the pattern of the wood fibers in most of the cases. In parallel, other signatures—this time more radiopaque—are visible as vertical lines in XR; in PuCT these become whiter as time elapses, suggesting that they have been filled during restoration, and probably involve only the upper layers. In the area of the green curtain, painted mainly with copper green, they are also visible in the MA-XRF distribution map of copper, due to the slightly lower signal of this element.

The combination of MA-XRF, XR, and IR reflectography was particularly effective for studying the preparatory phases of the painting, such as the ground layer, engravings, imprimitura, and underdrawing. The results of the OM and SEM/EDS analyses on a multi-layered micro-sample of the Virgin’s blue mantle allowed for a precise comparison for the confirmation of what was suggested by the non-invasive imaging techniques. Particularly, MA-XRF helped to detect the presence of calcium-, lead- and iron-based pigments on the entire painting and to attribute them to the preparatory phases, although it did not allow for a stratigraphic distinction between the ground layers—made of plaster and animal glue—and the thin imprimitura layer made of lead white and ochres. Interestingly, XR aided in recognizing, in some areas of the painting, the radiopaque brushstrokes of the imprimitura and also, at high magnifications, the small, more radiopaque imprimitura inclusions in the plaster layer, due to the opening and filling of the bubbles during the smoothing of the plaster layer: the average size of the inclusions turned out to be compatible with the one detected and sampled in the cross section. Moreover, the combination of the two X-ray techniques helped in studying the signs of the metal point engravings in correspondence to the figures’ haloes made using a drawing compass, also suggesting that they were probably drawn on the plaster layer.

Lastly, the presence of limited punched dots and a complete underdrawing, made with black ink and a brush, was clearly defined and studied through IR reflectography: the combination with XR helped in understanding the process of carrying out the preparatory phases of the painting.

All the collected data, especially those on the underdrawing, constitute an important opportunity and study tool for all the disciplines still involved in this research, as well as for a technical stylistic comparison with the original Raphael masterpiece displayed at the Alte Pinakothek in Munich, Germany.

## Figures and Tables

**Table 2:** Summary of the results on the preparation phases, from OM and SEM/EDS analyses on the cross section and MA-XRF, XR, and IRR imaging techniques on the whole painting.

Preparation Phases	OM and SEM/EDS	MA-XRF	XR	HR-SWIR
Ground layer	White layer, made of plaster	Detected the use of calcium-, lead-, and iron-based pigments for the preparation phases (ground and imprimitura not distinguishable between each other).	Not evident	Not evident
Imprimitura	Yellow-orange layer, made of lead white and ochres		Visible as radiopaque brushstrokes in some areas, also not in correspondence with lighter areas of paint; imprimitura inclusions in the plaster layer are visible at high magnification as a widespread presence of small, irregular white spots.	Not evident
Underdrawing	Not present in this sample	Not detectable	Visible in correspondence to the previously engraved haloes of the figures.	Limited punched dots and a complete underdrawing made with black ink and brush are visible.
Engravings	Hardly detectable with this technique; not present in this sample	Evident as a footprint in the Ca-, Pb-, and Fe-distribution maps (more intense signal in the Pb-distribution map; less intense signal in the Ca- and Fe-distribution map).	Visible in correspondence to the figures’ haloes made with a drawing compass.	Not evident
